# Questions about horse spleen ferritin crossing the blood brain barrier via mouse transferrin receptor 1

**DOI:** 10.1007/s13238-017-0481-8

**Published:** 2017-10-09

**Authors:** Kelong Fan, Meng Zhou, Xiyun Yan

**Affiliations:** 1 Key Laboratory of Protein and Peptide Pharmaceuticals, CAS-University of Tokyo Joint Laboratory of Structural Virology and Immunology, Institute of Biophysics, Chinese Academy of Sciences, Beijing, 100101 China; 2 University of Chinese Academy of Sciences, Beijing, 100049 China

Ferritin, an iron storage protein naturally occurring in the body, has emerged as a promising nanocarrier thanks to its unique architecture, excellent biocompatibility, and ability to self-assemble/disassemble (Fan et al., 2013). More specifically, the finding that human H-ferritin intrinsically targets tumor cells via binding to its receptor transferrin receptor 1 (TfR1) (Li et al., 2010; Fan et al., 2012; Liang et al., 2014; Zhao et al., 2016) inspired research into using ferritins for tumor target therapy.

Recently, a paper published in *Molecular Pharmaceutics* showed that horse spleen ferritin (HosFn) binds to and crosses the mouse BBB because of binding to mouse TfR1 (Chen et al., 2017), which raises great concerns to us.

To the best of our knowledge, HosFn lacks the ability to cross the mouse BBB, due to its lack of binding to mouse TfR1. Below we briefly describe others’ and our own evidence supporting such a conclusion.

Firstly, in our own experiments, we failed to detect any specific binding sites for HosFn on mouse BBB endothelial bEnd.3 cells.

In their paper, Chen et al. employed antibody-blocking experiments to show that the specific binding of HosFn to TfR1 on mouse bEnd.3 cells (Chen et al., 2017). However, the antibody used was mouse anti-human TfR1 (Clone M-A712; BD Bioscience), which was developed to specifically detect human TfR1 according to the Product Information provided by the manufacturer (BD Bioscience). Our ELISA assay confirmed that this antibody recognizes human TfR1, but not mouse TfR1 (Fig. 1A). We also demonstrated that although mouse TfR1 was abundantly expressed on bEnd.3 cells (Fig. 1C), mouse anti-human TfR1 antibody fail to interact with these cells (Fig. 1B), further indicating the idea that this antibody does not bind to mouse TfR1. Therefore, it appears impossible to block the binding of HosFn to mouse TfR1 with the antibody used in Chen et al.’s paper.

**Figure 1:**
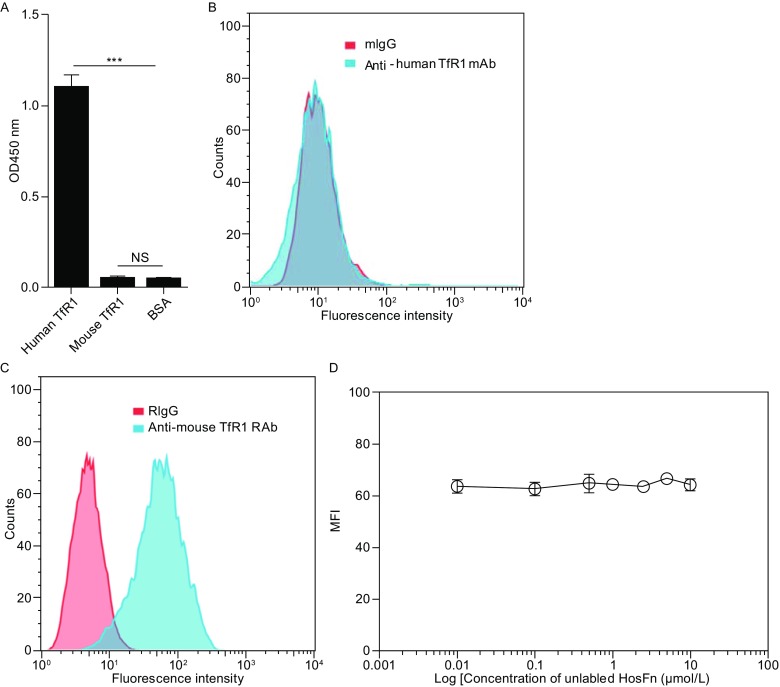
Lack of interaction between HosFn and mouse BBB endothelial bEnd.3 cells. (A) ELISA analysis of the binding of mouse anti-human TfR1 mAbs (Clone M-A712; BD Bioscience) to human TfR1 (11020-H07H, Sino Biological) and mouse TfR1 (50741-M07H, Sino Biological). (B) Flow cytometry analysis of the binding of mouse anti-human TfR1 antibody (Clone M-A712; BD Bioscience) to bEnd.3 cells. (C) Flow cytometry analysis of the binding of rabbit anti-mouse TfR1 antibody (50741-T16; Sino Biological) to bEnd.3 cells. (D) Competition for the binding sites of FITC-labeled HosFn (0.1 μmol/L) on bEnd.3 cells by unlabeled HosFn

In our experiments, the pre-incubation of bEnd.3 cells with excess amount (up to 100-fold mole excess) of unlabeled HosFn exhibited little effect on the binding of FITC-labeled HosFn to these cells (Fig. 1D). These results suggest that no specific binding sites for HosFn were present on bEnd.3 cells, which further confirmed that HosFn does not bind to mouse TfR1.

Secondly, the conclusion that HosFn cannot bind to and cross the BBB in mice was also drawn by Connor and colleagues (Fisher et al., 2007). The receptor of HosFn was reported to be the mouse L-ferritin receptor—mouse Scara 5 by different groups (Sun et al., 2011; Mendes-Jorge et al., 2014; Conti et al., 2016), which is also consistent with the fact that nearly 92% (22/24) composition of HosFn is L subunits (Harrison, 1986; Sun et al., 2011), and HosFn is typically regarded as L-ferritin in most previous research (Sun et al., 2011; Mendes-Jorge et al., 2014; Geninatti Crich et al., 2015; Conti et al., 2016).

Taken together, the conclusions of Chen et al.’s work need to be reconsidered, as they are misleading at best. Here, we provide more information about ferritins and their receptors. Importantly, we hope that this commentary will clarify the precise nature of the interactions of ferritins with their receptors.

To date, several ferritin receptors have been identified (Fan et al., 2013; Heger et al., 2014; Belletti et al., 2017). Although ferritins are highly conserved in various species, their receptors could be very different, e.g., the receptor of human H-ferritin is TfR1, while that of mouse H-ferritin is TIM-2. Thus, we cannot infer that different ferritin receptors function similarly. As a matter of fact, ferritins from different species do play contrasting roles beyond iron storage. We hope to point out here that when employing ferritin as nanocarriers to develop anti-disease system, people must clearly recognize which ferritin and the corresponding receptor are suitable for their purpose. When HosFn is chosen, its corresponding cross-interactive receptor is Scara 5 in mouse. Other suitable receptors for this purpose are yet to be determined.

## Electronic supplementary material

Below is the link to the electronic supplementary material.

Supplementary material 1 (PDF 43 kb)
