# Supplementary material for: Questions about horse spleen ferritin crossing the blood brain barrier via mouse transferrin receptor 1
Source: Protein Cell. 2017 Oct 9;8(11):788–90. doi: 10.1007/s13238-017-0481-8 (PMC5676598; doi:10.1007/s13238-017-0481-8)
Supplement: Supplementary file 1 — Supplementary material 1 (PDF 43 kb) [file 13238_2017_481_MOESM1_ESM.pdf]

Materials and Methods

Cellular culture

The mouse brain endothelial cells bEnd.3 were obtained from ATCC. The bEnd.3 cells were cultured in DMEM medium (Sigma-Aldrich) containing 10 % fetal calf serum (Sigma-Aldrich), penicillin (100 U/mL, Sigma-Aldrich) and streptomycin (100 μg/mL, Sigma-Aldrich) at 37 ℃ with 5 % CO_2.

ELISA assay:

96-well microplates were coated with human TfR1 (11020-H07H, Sino Biological), mouse TfR1 (50741-M07H, Sino Biological) or BSA (B2064, Sigma-Aldrich) of 10 μg/ml overnight at 4 ℃. The plates were then blocked with 5 % milk at 37 ℃ for 3 hours. After aspirated and washed with PBST and PBS, the plates were incubated with detection antibody (mouse anti-human TfR1 antibody, Clone M-A712, BD Bioscience) of 1 μg/ml at 37 ℃ for 1 hour. The plates were then incubated with the HRP-conjugated antibody (NA931V, GE Healthcare) at 37 ℃ for 45 mins. At last, the plates were added with TMB substrates and read the optical density at 450 nm.

Labeling of HosFn

The preparation of FITC-HFn was according to the report by Fisher et al. (Fisher et al., 2007). In brief, 200 nM fluorescein isothiocyanate isomer I (FITC; Sigma-Aldrich) was added to 50 nM HosFn (A3641, Sigma-Aldrich) solution in 1 mL carbonate/bicarbonate buffer (100 mM carbonate, pH 9.0). The mixture was incubated at room temperature for 1 hour. The mixture was purified with a PD MiniTrap G-25 column (GE Healthcare). The FITC-conjugated HosFn was concentrated, and buffer was exchanged with PBS in a Vivaspin-4 Centrifugal Concentrator (MWCO 100 k Da, Sartorius).

Binding and competing experiments:

For the binding assay, 100 μL detached bEnd.3 cells (2.5×10^6 cells/mL) were incubated with the mouse anti-human antibody (Clone M-A712, BD Bioscience) or the rabbit anti-mouse TfR1 antibody (50741-M07H, Sino Biological) of 1 μg/ml at 4 ℃ for 1 hour. Then the cells were incubated with Alexa Fluor 488®-labeled secondary antibody and finally analyzed by FACSCalibur flow cytometry system.

The competing experiments were performed according to the report of Fisher et al. (Fisher et al., 2007). Briefly, a total of 2.5×10^5 bEnd.3 cells were pre-incubated with increasing concentrations (0.01 μM to 10 μM) of unlabeled HosFn proteins prior to

incubation with 0.1 μM FITC-HosFn. After incubation, the cells were washed three times in cold PBS and collected. Cell-bound fluorescence was measured by FACSCalibur flow cytometry system.

References:

Fisher, J., Devraj, K., Ingram, J., Slagle-Webb, B., Madhankumar, A.B., Liu, X., Klinger, M., Simpson, I.A., and Connor, J.R. (2007). Ferritin: a novel mechanism for delivery of iron to the brain and other organs. Am J Physiol Cell Physiol 293, C641-649.
